# A Prevalence Study on *Anoplocephala* spp. in Serbian Horses: Navigating Diagnostic Challenges and Understanding Infection Risks

**DOI:** 10.3390/ani15142094

**Published:** 2025-07-16

**Authors:** Tijana Kukurić, Mihajlo Erdeljan, Jacqueline B. Matthews, Kirsty L. Lightbody, Corrine J. Austin, Natalia Peczak, Aleksandra Uzelac, Ivana Klun, Stanislav Simin

**Affiliations:** 1 Department of Veterinary Medicine, Faculty of Agriculture, University of Novi Sad, Trg Dositeja Obradovića 8, 21 000 Novi Sad, Serbia; tijana.kukuric@gmail.com (T.K.); erdeljanm@gmail.com (M.E.); 2 Austin Davis Biologics Ltd., Acorn Industrial Estate, Islip, Kettering NN14 3FD, UK; jacqui.matthews@austindavis.co.uk (J.B.M.); kirsty.lightbody@austindavis.co.uk (K.L.L.); corrine.austin@austindavis.co.uk (C.J.A.); natalia.peczak@austindavis.co.uk (N.P.); 3 Institute for Medical Research, Institute of National Importance for the Republic of Serbia, Bulevar Oslobođenja 18, 11 000 Belgrade, Serbia; aleksandra.uzelac@imi.bg.ac.rs (A.U.); iklun@imi.bg.ac.rs (I.K.)

**Keywords:** equine, tapeworm, coprology, serology, risk factors, Serbia

## Abstract

*Anoplocephala* spp. are common equine tapeworm species in Europe, frequently found in grazing horses. *Anoplocephala perfoliata* is the most pathogenic, clinically significant species associated with gastrointestinal disorders, particularly colic, and can have a fatal outcome in some horses. The aim of this study was to determine the infection prevalence of *Anoplocephala* spp. in Serbia and to identify relevant risk factors. A total of 173 horses from various regions were tested using a combination of diagnostic methods: coprological analysis via combined sedimentation–flotation and the Mini-FLOTAC technique, as well as serological testing using a commercial ELISA test. The overall prevalence was 38.7%, with a higher number of positive cases being identified by serology. It was demonstrated that coprology and serology are complementary approaches for prevalence studies. Infection risk factors included high stocking density, free-roaming status and co-infection with nematodes, while lower rainfall and temperate climate were associated with reduced risk of tapeworm infection. These findings highlight the challenges of *Anoplocephala* spp. detection and provide insight into the epidemiology of equine tapeworm infection in Southeastern Europe.

## Simple Summary

This study investigates the prevalence and risk factors for infection with equine tapeworms, specifically *Anoplocephala* spp., in a population of 173 horses across 15 districts in Serbia. Utilising a comprehensive diagnostic approach that combined coprological and serological methods, the overall tapeworm prevalence was 38.7%. Stocking density, horse use and concurrent nematode infection were shown to be risk factors associated with tapeworm infection, while lower rainfall and temperate climate were factors associated with reduced infection risk. These findings provide valuable insights into the epidemiology of *Anoplocephala* spp. infection in grazing horses in Southeastern Europe and new data for the development of education programmes for horse breeders and owners based on specific risk factors, infection control and prevention.

## 1. Introduction

Equine tapeworms (Cyclophyllidea: Anoplocephalidae) are frequent parasites of pasture-kept horses worldwide. Pathogenic *Anoplocephala perfoliata* is the most prevalent and clinically significant species, while *Anoplocephala magna* and *Anoplocephaloides mamillana* can also inhabit the digestive tract of horses [1,2,3,4,5,6]. Horses typically become infected by inadvertently ingesting the intermediate host, a nonparasitic oribatid mite, which is commonly found in soil. Cysticercoids from mites develop within the lumen of the horse’s small intestine and caecum into adult tapeworms [7]. Infected horses may exhibit colic, with pathological changes occurring at the site of infection, leading to complications such as intestinal obstruction, ileo-caecal or caeco-caecal intussusception, caecal perforation and peritonitis, which can be fatal [8,9,10]. Despite the pathogenicity of tapeworms, infection can be asymptomatic or clinical signs may not always be immediately apparent or even specific. In some cases, poor body condition may be the only noticeable, albeit unspecific, sign of infestation. Therefore, diagnosing anoplocephalidosis based solely on clinical examination is challenging [9].

Diagnosis of equine tapeworm infections historically relied on the detection of tapeworm eggs in faeces. However, the sensitivity of coprological methods is low, due to the intermittent release of egg-containing proglottids from adult worms, the presence of a significant number of immature or sterile worms as well as the uneven distribution and low quantity of tapeworm eggs in horse faeces [6,11,12]. To improve sensitivity, tests such as ELISA-based antibody detection in serum or saliva samples have been developed for tapeworm diagnosis [13]. Serological diagnosis is based on identifying IgG(T) antibodies against 12/13 kDa excretory/secretory antigens of *A. perfoliata*, with antibody levels correlating with the intensity of the infection [6,10,11,13,14].

Infection prevalence with equine tapeworms has been reported in different studies in several European countries, utilising different detection methods, including coproscopy or serology [5,6,10,15,16,17,18,19,20,21,22,23]. However, a comprehensive study of the prevalence of *Anoplocephala* spp. infection in Serbia has not been conducted to date. A few reports on the occurrence of tapeworm infection in horses from Serbia have been published, but the reported prevalence could be underrepresented due to limitations such as horse keeping practice, the focus on one isolated geographical region and the use of coprological methods that are not sensitive for tapeworm egg detection [24,25,26]. In addition, none of the studies analysed possible risk factors for infection, which is important to increase the epidemiological knowledge regarding *Anoplocephala* spp. in Serbia and Southeastern Europe.

Regarding therapeutic management, the situation in Serbia is particularly challenging. According to the official website of the Agency for Medicines and Medical Devices of Serbia (ALIMS) [27], the only anthelmintics registered for use in horses are ivermectin and fenbendazole, neither of which is effective against tapeworms. This lack of approved tapeworm-specific therapeutics highlights a major obstacle in the effective control of equine tapeworm infections in this country.

As pasturing is a common practice in Serbia, and therapeutics effective against tapeworms are not registered for commercial use, we hypothesised that the true prevalence of *Anoplocephala* spp. infection remains largely unknown.

The objective of the present study was to determine the prevalence of *Anoplocephala* spp. infection in a representative sample of the grazing horse population in Serbia using different diagnostic approaches, and to collect epidemiological data to identify potential risk factors for infection in this region.

## 2. Materials and Methods

### 2.1. Study Area

Serbia’s territory consists of low-lying regions in the northern province of Vojvodina, and hilly and/or mountainous terrain in the central-eastern and western regions, characterised by slightly different climate profiles. In the North, the climate is continental, dry, with typically cold winters and hot summers. The average annual precipitation is estimated at around 400–700 mm. The climate in central-eastern and western regions is temperate and more humid. The western region is particularly mountainous and receives more precipitation (>900 mm) than the central-eastern region (600–800 mm) [28].

### 2.2. Sample Size and Collection of Blood and Faeces

To determine the prevalence of infection, the minimum required sample size of a representative herd was calculated to be 121, using OpenEpi software [29]. The calculation was based on the total number of horses in 2022, which was 12,000 [30], and an average prevalence of 8.7%, based on available published coprological reports from Europe and Serbia [5,6,15,16,17,18,19,20,21,22,23,24,25,26], for a 95% confidence level and a 5% margin of error.

Blood was sampled from *V. jugularis* into sterile serum-collection vacutainers and centrifuged at 2000× *g* for 10 min to isolate the serum, which was then stored in microcentrifuge tubes at −20 °C until serological analysis.

Faecal samples were collected directly from the rectum of the animals or immediately after defecation, ensuring that the samples taken did not come into contact with the soil. The samples were then transported to the laboratory in a portable cooling box and stored in the refrigerator at 4 °C until coprological analysis.

### 2.3. Epidemiologic Data

Prior to sampling, horse owners completed a questionnaire that gathered information regarding the animals, their pastures, husbandry characteristics and any tapeworm control measures (Table 1).

### 2.4. Parasite Detection

#### 2.4.1. Coprological Methods

Faecal samples were first examined for tapeworm eggs using the modified combined sedimentation–flotation technique [31]. Briefly, 15 g of faeces was mixed with 40 mL of tap water, homogenised and filtered through a tea strainer. The mixture was then divided into two 50 mL tubes and centrifuged at 400× *g* for 10 min. After discarding the supernatant, the sediment was mixed with Sheather’s sugar solution (specific gravity 1.27), homogenised, transferred into two 15 mL tubes and centrifuged again at 400× *g* for 10 min. Additional flotation solution was added to form a slight convex meniscus, and a coverslip (18 × 18 mm) was placed on each meniscus. After 30 min, the coverslips were transferred to microscope slides and examined at 100× magnification. The eggs observed under both coverslips were quantified.

As a supplementary coprological method, a Mini-FLOTAC device was used. Eggs of other parasite species were also identified and recorded. For this procedure, 5 g of faeces was mixed with 45 mL of NaCl solution (specific gravity 1.2) using a Fill-FLOTAC sample preparation kit [32]. The chambers of the reading disc were filled, and raw parasite egg counts were recorded after 10 min of passive flotation. The total number of eggs for different ova types was multiplied by five to calculate the faecal egg counts (FECs) for each type.

Co-infection status (i.e., detection of strongyle and tapeworm eggs in faecal samples) was assessed based on the coprological results obtained using both the combined sedimentation–flotation technique and the Mini-FLOTAC method.

#### 2.4.2. Serology

Sera were analysed for the presence of IgG(T) antibodies using the Tapeworm Blood Test ELISA which can detect *A. perfoliata* infection (Austin Davis Biologics Ltd., Northamptonshire, UK) [13]. The test was performed at Austin Davis Biologics laboratories using the methodology conducted in the commercial service (https://www.austindavis.co.uk/tapeworm-blood-testing-service, accessed on 14 July 2025) based on Lightbody et al. (2016) [13]. Briefly, 100 μL of diluted serum and purified horse IgG calibration standards were applied in duplicate onto plate wells coated with 12/13 kDa tapeworm antigen and incubated. Goat anti-horse IgG(T) antibody conjugated to horseradish peroxidase was used for detection. The reaction was stopped, and the absorbance was read at 450 nm. The negative control (sample buffer) absorbance was subtracted from sample and calibrator readings, the standard curve slope was calculated, and the results were reported as specific IgG(T) antibody-binding serum scores. Tapeworm serum scores were reported as low (<2.70), borderline (2.70 to 6.30) and moderate/high (>6.30) categories. In this study, both borderline and moderate/high categories were deemed indicative of tapeworm infection. However, it is important to note that, according to the validation studies [13], a few horses with low levels of tapeworm infection may fall within the low category.

### 2.5. Statistical Analyses

The prevalence of *Anoplocephala* spp. in horses was calculated for both coprological and serological methods, along with Stern’s or Wald’s 95% confidence intervals (CIs), using Quantitative Parasitology 3.0 [33]. Concordance between sedimentation–flotation and Mini-FLOTAC was determined using Cohen’s kappa statistics (interrater reliability test); κ values of <0.00 indicated poor, 0.00–0.20 none, 0.21–0.39 minimal, 0.40–0.59 weak, 0.60–0.79 moderate, 0.80–0.90 strong and >0.90 almost perfect agreement [34]. In contrast, the difference between coprological and serological test results was assessed using McNemar’s test for paired proportions.

For risk factor analysis, horses were considered to be infected if either eggs were detected by coprology or the serology result was reported as borderline or moderate/high (i.e., serum score ≥ 2.70). For independent dummy variables, the reference category was the one with the highest frequency of observations, except for the variable ‘stocking density’, where category levels were arranged from ideal to inadequate (Table 1). For the only dichotomous variable ‘presence of other parasites’, the category ‘no’ was used as reference. Univariate logistic regression was performed, and variables significant at the *p* ≤ 0.15 level were tested for collinearity by using Cramer’s phi measure of association (with values of ≥0.67 denoting high collinearity), and included in the multivariate models to analyse the influence of examined factors as independent categorical variables on *Anoplocephala* spp. infection. The overall fit of the models was tested with Hosmer–Lemeshow (HL) goodness-of-fit statistics. Final models were obtained by the forward or backward stepwise, likelihood ratio or Wald method, as appropriate for the best fit. The results are presented as adjusted odds ratios (OR) with 95% CIs. The level of significance was 5%.

Risk factor analysis, test concordance (kappa statistics), McNemar’s test (for paired proportions) and descriptive statistics (for egg counts) were performed using the SPSS version 11.5 statistical package (SPSS Inc., Chicago, IL, USA). In addition, 95% CIs were calculated.

## 3. Results

**Figure 1:**
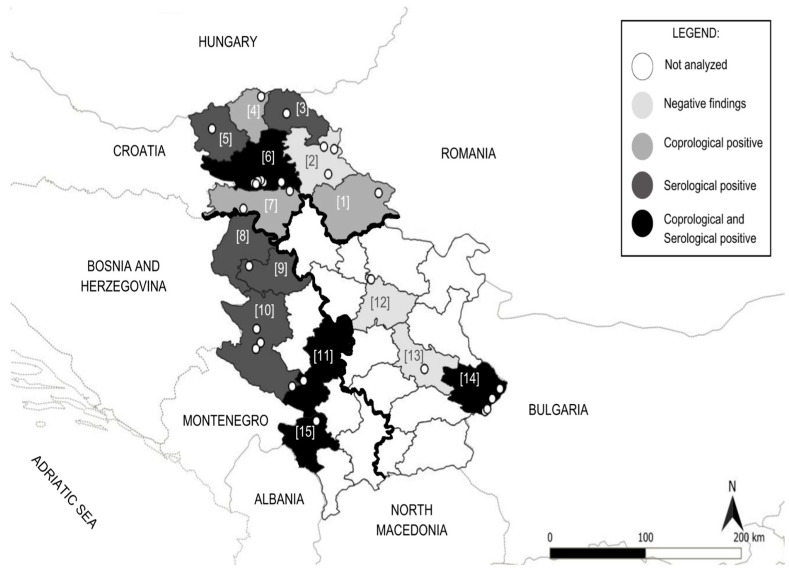
Map of Serbia showing municipal districts (numbered) and sampling locations (open circles). Territorial divisions into three regions: Northern Serbia, central and southeastern Serbia and western Serbia, based on climate and humidity, are represented by thick black lines. The municipal districts are coloured based on the findings: light grey (negative by serology and coprology: 2, 12, 13), grey (positive by coprology: 1, 4, 7), dark grey (positive by serology: 3, 5, 8, 9, 10) and black (positive by both methods: 6, 11, 14, 15). White represents districts from which the samples were not collected.

A total of 173 blood and faecal samples were collected from grazing horses, covering representative terrain and climate conditions across Serbia (29 localities) between September 2022 and November 2023 (Figure 1). The estimated overall prevalence of infection with *Anoplocephala* spp. was 38.7% (67/173; 95% CI: 31.7–46.2), based on combined findings by coprology and serology. Altogether, 38 different horse owners completed the questionnaires. The examined population consisted of 14 horse breeds (Bosnian Mountain Horse, Lipizzaner, Nonius, Holsteiner, Oldenburger, Friesian, French Trotter, Standardbred, English Thoroughbred, Haflinger, Arabian Horse, Posavac, Appaloosa and Pony), one group of grade horses and two groups of half-blood horses (English Half-blood and Zobnatica Half-blood). The most represented breed was the Bosnian Mountain Horse, accounting for 31.2% (54/173) of the population.

Information on anthelmintic use revealed that owners typically administered ivermectin, and occasionally fenbendazole or pyrantel, in the form of paste, granules or liquid. Off-label use of injectable ivermectin applied orally was also reported. None of the horses were treated with praziquantel. Regarding the most recent treatment specifically, ivermectin was administered in 120/173 (69.4%) horses, fenbendazole in 6/173 (3.5%) horses, while 47/173 (27.1%) had not received any anthelmintic treatment.

### 3.1. Prevalence of Anoplocephala spp. Infection by Coprology

**Figure 2:**
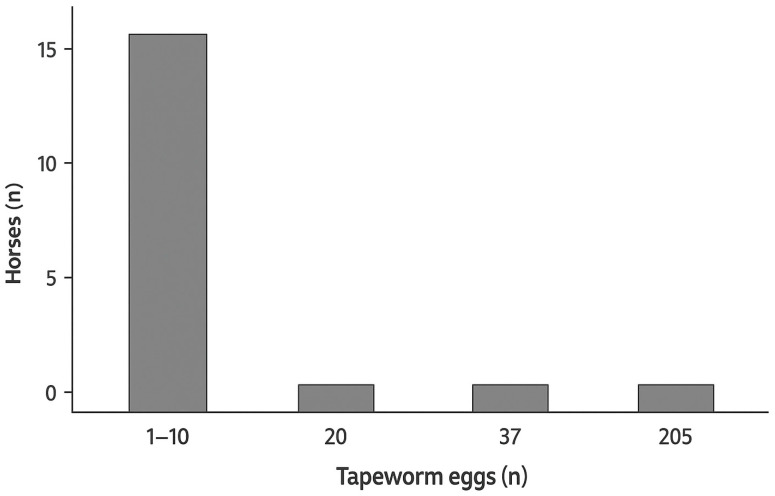
Distribution of tapeworm egg counts (sedimentation–flotation) in n = 19 horses.

Eggs with morphological features characteristic of Anoplocephalidae were detected in 11% of faecal samples (19/173; 95% CI: 6.97–16.7) by sedimentation–flotation. *Anoplocephala* spp. eggs were counted on two coverslips per sample. The number of tapeworm eggs varied significantly, ranging from 1 to 205, with a mean of 18.74 ± 45.82 and a median of 7. In the majority of samples, no more than ten tapeworm eggs were present (mode = 8; Figure 2). Of these 19 samples, only three were also positive using the Mini-FLOTAC (1.7%; 3/173; 95% CI: 0.5–5.1) with faecal egg counts of 5, 20 and 115 EPG. Minimal agreement (Cohen’s *κ* = 0.25, *p* < 0.05) was observed between sedimentation–flotation and Mini-FLOTAC. Additionally, the presence of other equine parasite eggs was noted, such as strongyles, *Parascaris* spp., *Oxyuris equi* and *Strongyloides westeri*.

### 3.2. Seroprevalence of Tapeworm Infection

The tapeworm ELISA detected specific antibodies in 35.3% of examined horse sera (61/173; 95% CI: 28.3–42.8). Of these, 17.9% (31/173) were diagnosed as borderline and 17.3% (30/173) as moderate/high.

### 3.3. Comparison of Both Detection Methods

Although more infections were diagnosed by serology as opposed to coprology (Table 2), tapeworm eggs were detected in six horses with low serum scores. The difference between coprological and serological test results was not statistically significant according to McNemar’s test (*p* = 0.080), although serology identified more positive cases than coprology.

### 3.4. Risk Factor Analysis

Positive findings by coprology and serology were considered for risk factor analysis. Plausible factors included as variables for univariate analysis are shown in Table 3. The following were associated with *Anoplocephala* spp. infection: age, sampling period, breed, use, type of husbandry, stocking density, period spent at pasture, multispecies pasturing, presence of other parasites (as detected by coprology) and region.

However, collinearity testing of the independent variables determined a high Cramer’s phi value of 0.679 for ‘use’ vs. ‘type of husbandry’, and of 0.681 for ‘stocking density’ vs. ‘multispecies pasturing’, which is why it was decided not to include them in multivariate models simultaneously. In addition, multicollinearity was observed for ‘breed’ in relation to several variables, as well as for ‘period at pasture’. Therefore, it was decided not to use ‘breed’ and ‘period at pasture’ in the multivariate analyses, and to build multivariate models by alternately using variables ‘use’ and ‘type of husbandry’, as well as ‘stocking density’ and ‘multispecies pasturing’.

Thus, the following variables were included in the multivariate models: age group, sampling period, use (or type of husbandry), stocking density (or multispecies pasturing), infection with other parasites and region. The final model (Table 4) was selected based on the most favourable value of the HL statistic (0.831), obtained by excluding variables (i.e., using the backwards stepwise method) from the model which utilised the ‘use’ as well as ‘stocking density’ variables. It was shown that infection with *Anoplocephala* spp. is independently influenced by use, stocking density, infection with other parasites and region. The likelihood of infection in free-roaming horses was 13-fold higher as opposed to that in horses used for recreational riding. Also, horses kept in conditions of borderline (0.17–0.3 ha/horse) or inadequate (≤0.1 ha/horse) stocking density were at almost 6-fold higher and 11-fold higher risk, respectively, than those pastured in ideal conditions of more than 0.8 ha/horse. Horses infected with nematodes (as detected by coprology) had a 15-fold higher risk of being infected with tapeworms. Horses from central-eastern Serbia were at 79% lower estimated risk of infection compared to those from the northern region.

## 4. Discussion

This study represents the first comprehensive prevalence assessment of *Anoplocephala* spp. infection in horses in Serbia, utilising several detection approaches—direct: egg detection with combined sedimentation–flotation and Mini-FLOTAC, and indirect: detection of tapeworm-specific IgG(T) antibodies. The presence of equine tapeworms has been established by previous parasitological studies in Serbia. However, due to their limitations, such as horse keeping practices, a narrow geographical focus and the use of coprological methods that are not sensitive for tapeworm egg detection, further investigation was warranted. Results of these studies, based on coprology, reported the prevalence ranging from 0.91% to 38.46% [24,25,26]. The prevalence of equine tapeworms based on coprological diagnosis varies considerably across European countries. Generally low values have been reported in several regions, including Macedonia and Greece (0.4%), Greece alone (8.5%), Hungary (0.6%), Croatia (1%) and Albania (3.8%) [16,17,19,20,23]. Slightly higher prevalence was observed in Romania (6.6%) and Bulgaria (14%) [15,22], while in Central Europe, values such as 1.99% in Slovakia [6], 10.4% in Germany and Austria [18], 14.3% in Germany alone [21] and 3.7% in Italy [5] have been reported. In studies from Germany and Slovakia, where both coprological and serological methods were applied, seroprevalence ranged from 16.2% to 56.95%, revealing a substantially higher number of positive horses compared to coprology alone [6,10]—similar to the results presented here. This study shows that the prevalence based on coprology using a tapeworm-specific combined sedimentation–flotation technique was 11% in Serbian horses. The obtained coprological prevalence is close to the anticipated prevalence of 8.7%, which was calculated as the average value based on coprological studies conducted in Europe and Serbia. In this study, the combined sedimentation–flotation technique using a sugar solution and 15 g of faeces, as described previously [31], was used to detect tapeworm eggs, while the Mini-FLOTAC method was used to determine the presence of tapeworm and other parasite eggs. It has been reported that faecal egg count methods (such as Mini-FLOTAC) exhibit high specificity (90–100%) but low sensitivity in detecting tapeworm infections, which is associated with the uneven release and distribution of eggs, as well as with the use of a smaller amount of faecal sample [12]. The results of this study are in concordance with these findings, as only faecal samples from three of the 19 horses with *Anoplocephala* spp. eggs detected, as determined by combined sedimentation–flotation, were confirmed to contain eggs using the Mini-FLOTAC device. Cohen’s kappa coefficient of 0.25 indicates only minimal agreement between the two coprological methods. As shown in Table 2, the number of *Anoplocephala* spp. eggs was highly variable, and furthermore, in the authors’ experience, the same faecal sample did not consistently yield eggs, which is in consistent with other reports [8,12]. However, recent studies from Germany [10] and Slovakia [6] demonstrated a notable difference in detection rates between sedimentation–flotation and Mini-FLOTAC methods applied to the same samples, with positivity rates of 0.6% and 1.99%, respectively. Similarly, a recent study reported that Mini-FLOTAC detected only 16% of infections, compared to 70% using the Proudman & Edwards sedimentation–flotation method, further highlighting the inferior sensitivity of Mini-FLOTAC for diagnosing *Anoplocephala* spp. infection [35]. Together, these findings suggest that applying multiple coprological techniques can improve diagnostic sensitivity and increase the likelihood of detecting infected horses.

To overcome limits inherent in coprology [36], a commercially available tapeworm ELISA was also used in this study for serological diagnosis of infection. The 96-well plate-based setup facilitates the simultaneous screening of numerous samples, making it ideal for identifying geographical regions where control measures should be implemented. This approach enables specific recommendations to be made to breeders and horse owners regarding husbandry practices and anti-cestode treatment. The absence of cross-reactivity of the 12/13 kDa E/S antigen of *Anoplocephala* spp. with other common parasites of horses, such as nematodes, *A. mamillana* and fly larvae has been confirmed [37,38]. One previous study [1] reported a degree of antigenic cross-reactivity in the region of 12–13 kDa in *A. perfoliata* excretory/secretory products in some *A. magna* infected horses; however, the treatment history of the assessed horses was unknown, so it could not be determined whether the binding antibody was associated with previous infection with *A. perfoliata*. Furthermore, antibody reactivity to *A. magna* antigens was not assessed in horses infected with *A. perfoliata*. Therefore, the ability of this test to discriminate between *A. perfoliata* and *A. magna* remains unknown.

The level of tapeworm-specific IgG(T) measured in the ELISA has been reported to correlate with *A. perfoliata* burden, which allows for the identification of horses with potentially pathogenic burdens (>20 tapeworms) which may be more at risk of developing ileocecal lesions [39]. According to the current findings, the seroprevalence of infection was 35.3%, with nearly identical numbers of horses in the borderline and moderate/high categories. Based on the results herein, correlations with tapeworm burden could not be ascertained as worm enumerations were not performed, the egg counts were highly variable and did not always correlate with the serology results and the owners/breeders did not report any clinical symptoms of tapeworm-associated disease. One limitation of the tapeworm ELISA test is the half-life of 21 [40] and 35 days [41] of IgG(T) in blood. This is a confounding factor when trying to ascertain active infection in horses which are grazed on contaminated pasture, and the likelihood of re-infection is high, but insufficient time has elapsed since the last anthelmintic treatment that would enable discrimination between previous and current infection [13,37,42,43]. For this reason, the test’s guidelines stipulate that horses should not be tested within 4 months of cestocidal treatment. These guidelines were followed in the current study; none of the horses had received a cestocidal anthelmintic in the preceding 4 months, so the 35.3% prevalence reported by serology is unlikely to have been confounded by recent treatment effects. Indeed, the relatively high prevalence of *A. perfoliata* infection reported underscores the urgent need for the registration and availability of cestocidal anthelmintics for use in Serbian horses to mitigate the potential clinical impact of these parasites. In regions with a high prevalence of *A. perfoliata*, infections and hence, antigen-specific IgG(T), may persist year-round in some horses on contaminated paddocks. However, improvements in management (such as reducing stocking density, resting paddocks and/or regularly removing faeces) can reduce pasture infectivity and, in these situations, antibody testing can be used to demonstrate reductions in infection levels as well as inform the need to treat those horses which are more susceptible to re-infection despite lower contamination [44].

In the present study, tapeworm eggs were detected coprologically in six horses that tested low in the tapeworm ELISA. Given the known low sensitivity and low genus-level specificity of coprological techniques for detection, it is not yet known whether all eggs observed were of the species *A. perfoliata*. This discrepancy can also, in part, be explained by the known sensitivity and specificity of the ELISA used here, which are 85.2% and 77.6%, respectively, based on a threshold of ≥1 tapeworm [13]. At this cut-off, the test accurately identified 100% of horses with over 20 tapeworms. However, approximately 15% of horses with low burdens (1–20 tapeworms) may be classified as seronegative. These horses might still show positive faecal egg counts, although their parasite burden is considered non-pathogenic based on validation data [13] of the ELISA test. Therefore, while the ELISA provides higher diagnostic sensitivity overall, combining serological and coprological methods is useful in prevalence studies wherein the requirement is to report the number of horses infected. Notably, the difference between coprological and serological test results in this study was not statistically significant according to McNemar’s test (*p* = 0.080), although serology identified more positive cases. This suggests that an integrated approach using both diagnostics may maximise detection of infection by compensating for the limitations of each test.

When it comes to risk factors, stocking density, concurrent nematode infection, horse use and climate were identified as the main risk factors for tapeworm infection in the horses assessed here (Table 4). High stocking density on pastures may contribute to the accumulation of high helminth burdens and, hence, the development of gastrointestinal disease, as horses are forced to graze near faecal piles, which facilitates parasite transmission [45]. Additionally, since some mite species are coprophagous, their numbers may also be higher in the vicinity of faeces [46]. In temperate climates, it is recommended to allocate 0.4 to 0.8 hectares per horse to maintain adequate pasture productivity [47,48]. In the present study, stocking density was found to be a significant risk factor for *Anoplocephala* spp. infection. Horses grazing on pastures with limited space—classified as borderline (0.17–0.3 ha/horse) or inadequate (≤0.1 ha/horse)—had a markedly higher risk of infection compared to those kept at a lower stocking density (>0.8 ha/horse). Specifically, horses kept under borderline conditions showed nearly a 6-fold increased risk, while those kept under inadequate conditions faced an approximately 11-fold higher risk. In addition, horses infected with nematodes (as detected by positive strongyle faecal egg counts) exhibited a 15-fold greater likelihood of concurrent tapeworm infection. These findings highlight the potential of nematode infection as an epidemiological indicator, particularly in herds with a known history of tapeworm presence. In such cases, a high prevalence of nematode infections may point to an increased risk of tapeworm infection, even when direct diagnostic methods such as coprological or serological testing yield negative results. According to the findings here, free-roaming (semi-wild) horses had a 13-fold higher risk of infection compared to those used for recreational riding, which is consistent with statistical data from published prevalence studies which show a higher occurrence of tapeworm infections in horses that had year-round unrestricted access to pasture [6,10,49]. This increased risk may also be associated with the fact that free-roaming horses are generally treated with anthelmintics far less frequently, or never, as indicated by the questionnaire responses collected in this study. In terms of the influence of climate and season on infection risk, lower rainfall, characteristic of central-eastern Serbia, was associated with a reduced risk of infection here. It has been reported that both the prevalence and intensity of *A. perfoliata* infections are highest in horses grazing in humid and marshy areas [9,50], conditions which occur in the northern region of Serbia. Additionally, the geographical distribution of *Anoplocephala* spp. in Europe, estimated through prevalence, suggests that the tapeworms are more common in Northern Europe as opposed to the Mediterranean region [49]. As to the seasonal impact on infection prevalence, the highest number of positive horses was recorded during summer, although some previous studies have reported a peak prevalence of *Anoplocephala* spp. infection in autumn and winter [51,52,53]. The increased number of infected horses during the summer is likely to be directly associated with the presence of intermediate hosts during the grazing season.

## 5. Conclusions

This study highlights a considerable prevalence of *Anoplocephala spp*. infection among horses in Serbia, particularly in free-roaming populations. The findings underscore the need for improved diagnostic awareness, targeted control programs and the registration of effective cestocidal treatments. In addition, strengthening education on pasture management and parasite surveillance is essential for sustainable parasite control, especially in light of the emerging issue of anthelmintic resistance in *A. perfoliata* [4].

## Figures and Tables

**Table 1:** Data collected on plausible epizootiological risk factors for *Anoplocephala* spp. infection in horses.

Factor	Categories/Responses
Gender	Mares; Stallions; Geldings
Age group	Foals (<1 year); Yearlings (≥1 and <2 years); Adolescent/Young adults (2–5 years); Adults (6–14 years); Seniors (≥15 years)
Season of sampling *	Fall; Winter; Spring; Summer
Type of husbandry	Pasture; Stable with run; Pasture and stable
Breed (type)	Bosnian Mountain; Grade; Lipizzaner; Other **
Type of use	Recreational riding; Free-roaming/semi-wild; Racing/sport; Carriage/parade horses
Herd size	Number of horses
Pasture size	Size in hectares
Stocking density ***	Ideal (>0.8 ha); Adequate/recommended (0.4–0.8 ha); Borderline (0.17–0.3 ha); Inadequate (≤0.1 ha)
Time spent on pasture	Seasonal (spring and summer); Year-round; Occasionally/sporadically
Multispecies pasturing	Yes; No; Yes, with donkeys
Presence of other parasites	No; Yes
Praziquantel treatment	No
Region ****	Northern Serbia; Central and Southeastern Serbia; Western Serbia

* Determined from date of sampling. ** Other represents less frequent breeds/types (≤15 horses), including Nonius, Holsteiner, Oldenburger, Friesian, French Trotter, Standardbred, English Thoroughbred, Haflinger, Arabian Horse, Posavac, Appaloosa, English Half-blood, Zobnatica Half-blood and Pony. *** Stocking density was calculated from herd and pasture size. **** Location was used to assign the region.

**Table 2:** Contingency table with coprology and serology results.

		Serology			Row Total
		Positive		Negative	
		moderate/high	borderline	low	
Coprology	Positive	8	5	6	19
	Negative	22	26	106	154
Column Total		61		112	173

**Table 3:** Detection of *Anoplocephala* spp. eggs in faeces and/or horses that were reported as borderline or moderate/high in the study group (n = 173) according to independent variable categories, and results of univariate logistic regression (n—number of animals; IP—confidence interval; OR—odds ratio).

Factor	n	Prevalence (%)	95% IP	OR	95% IP	*p*
Gender						
Mare	118	39.8	30.9–49.2	1.00		0.681
Stallion	42	36.4	23.8–50.4	1.37	0.68–2.79	
Gelding	13	0.00	0.00–24.7	0.00	0.00–NA	
Age group						
<1	4	75.0	19.4–99.4	1.00		**0.104**
1–2	8	25.0	3.2–65.1	0.11	0.01–1.78	
2–5	52	48.4	27.0–54.9	0.23	0.02–2.32	
6–14	85	43.5	32.8–54.7	0.26	0.03–2.57	
≥15 years	24	16.7	4.7–37.4	0.07	0.01–0.82	
Sampling period						
Autumn	72	31.9	24.4–44.0	1.00		**0.029**
Winter	27	40.7	22.4–61.2	1.46	0.59–3.65	
Spring	37	29.7	15.9–47.0	0.90	0.38–2.13	
Summer	37	59.5	42.1–75.2	3.12	1.37–7.11	
Breed						
Bosnian Mountain	54	74.1	60.3–85.0	1.00		**<0.001**
Grade	29	34.5	17.9–54.3	0.18	0.07–0.49	
Lipizzaner	26	15.4	4.4–34.9	0.06	0.02–0.22	
Other	64	20.3	11.3–32.2	0.09	0.04–0.21	
Use						
Recreational riding	70	24.3	14.8–36.0	1.00		**<0.001**
Free-roaming horses	62	75.8	63.3–85.8	9.77	4.40–21.69	
Sport horses	34	8.8	1.9–23.7	0.30	0.08–1.11	
Carriage/parade horses	7	0.00	0.00–41.0	0.00	0.00–NA	
Type of husbandry						
Pasture	79	68.3	56.9–78.4	1.00		**<0.001**
Stable with run	73	9.6	3.9–18.8	0.05	0.02–0.12	
Pasture and stable	21	28.6	11.3–52.2	0.18	0.06–0.53	
Stocking density						
Ideal	29	72.4	52.8–87.3	1.00		**<0.001**
Adequate/recommended	18	38.9	17.3–64.2	0.24	0.07–0.85	
Borderline	68	47.1	34.8–59.5	0.34	0.13–0.87	
Inadequate	58	12.1	5.0–23.3	0.05	0.02–0.16	
Period at pasture						
Seasonal (spring-summer)	111	20.7	13.6–29.4	1.00		**<0.001**
All year	59	74.6	61.6–85.0	11.22	5.33–23.63	
Sporadically	3	0.00	0.00–70.8	0.00	0.00–NA	
Multispecies pasturing						
Yes	68	33.8	22.8–46.3	1.00		**<0.001**
No	65	13.8	6.5–24.7	0.31	0.13–0.75	
Yes, with donkeys	40	87.5	73.2–95.8	13.70	4.73–39.66	
Infection with other parasites						
No	44	4.5	0.56–15.5	1.00		**<0.001**
Yes	129	50.4	41.4–59.3	21.33	4.95–91.83	
Region						
Northern	112	29.5	21.2–38.8	1.00		**<0.001**
Central-Eastern	25	28.0	12.1–49.4	0.93	0.35–2.44	
Western	36	75.0	57.8–87.9	7.18	3.05–16.92	
Total	173	38.7	31.4–46.4			

**Table 4:** Risk factors for infection with *Anoplocephala* spp. in horses (n = 173) in Serbia. Final multivariate model.

Factor	Adjusted OR	95% IP	*p*
Use			
Recreational riding	1.00		
Free-roaming horses	13.43	3.67–49.11	**<0.001**
Sport horses	0.25	0.05–1.42	0.118
Carriage/parade horses	0.00	0.00–NA	0.999
Stocking density			
Ideal	1.00		
Adequate/recommended	1.93	0.32–11.75	0.474
Borderline	5.80	1.07–31.56	**0.042**
Inadequate	10.80	1.10–106.40	**0.041**
Infection with other parasites			
No	1.00		
Yes	15.11	2.25–101.42	**0.005**
Region			
Northern	1.00		
Central-Eastern	0.21	0.05–0.93	**0.040**
Western	3.25	0.78–13.61	0.106

## Data Availability

The data used to support the findings of this study are available in the present manuscript.
